# Chief Digital Officer (CDO): Literaturanalyse und Handlungsempfehlungen für die Praxis

**DOI:** 10.1365/s40702-022-00864-x

**Published:** 2022-04-12

**Authors:** Anna Hermes, René Riedl

**Affiliations:** 1 Institut für Wirtschaftsinformatik – Information Engineering, Johannes Kepler Universität Linz, Altenberger Straße 69, 4040 Linz, Österreich; 2 Fakultät für Wirtschaft & Management, Abteilung für Digital Business, Fachhochschule Oberösterreich, Wehrgrabengasse 1–3, 4400 Steyr, Österreich

**Keywords:** Chief Digital Officer, CDO, Chief Information Officer, CIO, Digitalisierung, Digitale Transformation, Chief Digital Officer, CDO, Chief Information Officer, CIO, Digitalization, Digital Transformation

## Abstract

Um im Zeitalter der Digitalisierung wettbewerbsfähig zu bleiben und um die digitale Transformation sicherzustellen, schaffen Unternehmen zunehmend die Stelle eines Chief Digital Officer (CDO). Um Unternehmen bei Entscheidungen rund um die Ein- und Ausrichtung der Position des CDOs zu unterstützen, haben wir in folgenden Bereichen die Fachliteratur systematisch analysiert: (1) Gründe für eine CDO-Position, (2) Aufgaben des CDOs, (3) Anforderungen an einen CDO sowie (4) Abgrenzung zwischen CDO und Chief Information Officer (CIO). In der Fachliteratur wird insbesondere die strategische Führung bei Veränderungen als Grund für die Schaffung einer CDO-Position genannt. Damit einhergehend ist die Digitalisierung und das Implementieren der digitalen Transformation die am häufigsten genannte Aufgabe sowie das strategische und geschäftsorientierte Denken die am häufigsten genannte Anforderung. In der Abgrenzung des CDOs zum CIO wird der CDO primär als Stratege und der CIO in erster Linie als Verantwortlicher für die IT-Infrastruktur gesehen. Basierend auf den Ergebnissen der Literaturanalyse beschreiben wir Handlungsempfehlungen für Entscheidungsträger in der Praxis, die sich eine Übersicht über die möglichen Ein- und Ausrichtungen der CDO-Position schaffen wollen.

## Abstract (en)

In order to remain competitive in the age of digitalization, and to facilitate digital transformation in organizations, companies are increasingly creating the position of the Chief Digital Officer (CDO). To support companies in making decisions about the establishment and alignment of the CDO position, we have systematically analyzed the academic literature within the following areas: (1) reasons to hire a CDO, (2) CDO tasks, (3) CDO requirements, and (4) the CDO position vs. the Chief Information Officer (CIO) position. In the academic literature, the strategic management of changes is cited most frequently as the reason for hiring a CDO. In line with this, the digitalization and the implementation of digital transformation was the most frequently mentioned CDO task, and strategic and business-oriented thinking was the most frequently mentioned CDO requirement. Further, the CDO is predominantly seen as a strategist and the CIO primarily as the person responsible for the IT infrastructure. Based on the results of the literature analysis, we give recommendations for decision-makers in practice who want to get an overview of the possible establishment and alignment of the CDO position.

## Einleitung

Um im Zeitalter der Digitalisierung wettbewerbsfähig zu bleiben, sind die Initiativen eines Unternehmens zur digitalen Transformation von entscheidender Bedeutung (Hess et al. 2016; Verhoef et al. 2021). Darüber hinaus hat die COVID-19-Pandemie viele Unternehmen weltweit dazu gezwungen, Unternehmensabläufe zu digitalisieren (Van Looy 2021). Digitale Transformation kann wie folgt definiert werden: „a change in how a firm employs digital technologies, to develop a new digital business model that helps to create and appropriate more value for the firm“ (Verhoef et al. 2021, S. 889). Für Unternehmen ist somit die gezielte Einführung und Nutzung von neuen Technologien zur Wertsteigerung des Unternehmens unerlässlich (Verhoef et al. 2021). Betrachtet man zum Beispiel den Bereich des Einzelhandels (Stieninger et al. 2019; Scott et al. 2020; Sheth 2020), aber auch andere Bereiche wie den Banken- (Giebe 2019) oder Mediensektor (Hess et al. 2016), wird klar, dass Kunden zunehmend Services online nutzen. Daher ist es unerlässlich, digitale Technologien einzusetzen, um den Kundenmarkt bestmöglich zu erschließen und die Kundenerfahrung (Customer Experience, CX) zu optimieren (Grewal und Roggeveen 2020; Verhoef et al. 2021).

Nicht zuletzt vor den aktuellen Entwicklungen in Richtung eines noch weiter und rascher ansteigenden Digitalisierungsgrades in vielen Organisationen gewinnt die Position des Chief Digital Officers (CDOs)^1^, welcher die digitale Transformation im Unternehmen plant und umsetzt (Walchshofer und Riedl 2017), als Schlüsselfunktion im Top-Management an entscheidender Bedeutung (Alatovic et al. 2020). Weltweit schaffen Unternehmen mittlerweile die Stelle eines CDOs, um die digitale Transformation im Unternehmen voranzutreiben. Der CDO steht jedoch oft der Stelle des Chief Information Officers (CIOs) gegenüber (Walchshofer und Riedl 2017), da beide Positionen mit technischen und organisatorischen Themen im Unternehmen betraut sind. Vor dem Hintergrund dieser Entwicklungen soll die vorliegende systematische Literaturanalyse dazu beitragen, Entscheidungsträger in Unternehmen rund um die Ein- und Ausrichtung der CDO-Position und bei der Abgrenzung der CDO-Rolle von der Rolle des CIOs zu unterstützen.

In einer aktuellen Literaturanalyse hat sich Drechsler (2020) damit befasst, wie CDOs zum Unternehmenserfolg beitragen. Die Autorin hat unter anderem herausgearbeitet, dass sich auf individueller CDO-Ebene folgende Faktoren auf die Unternehmensperformance auswirken: Bildungs- und Berufserfahrungshintergrund sowie das Wissen, Eigenschaften und Fähigkeiten sowie die im Unternehmen ausgeführte CDO-Rolle und das damit einhergehende Verhalten des CDOs im Unternehmen. Diese Faktoren interagieren unter anderem mit der Beziehung zum Chief Executive Officer (CEO), internen Prozessen oder der Unternehmensstruktur und -strategie. Moker (2020) hat in einem Research-in-Progress-Artikel fünf Beiträge analysiert und fokussiert sich dabei auf folgende Bereiche: (1) CDO-Position (Einrichtung, strategischer Einfluss, Einordnung im Unternehmen), (2) CDO-Person (Persönlichkeit und Beziehung mit anderen Positionen), (3) Umwelt (Stellenwert des CDOs, Einfluss auf die Unternehmensperformance). Auch Kessel und Graf-Vlachy (2021) haben die CDO-Literatur analysiert und leiten ein konzeptionelles Modell für die CDO-Forschung ab. Dieses Modell beinhaltet Gründe für eine CDO-Präsenz, CDO-Typen und deren Beziehungen zu anderen Führungskräften und Mitarbeitern sowie Konsequenzen einer CDO-Position. Trotz des Umstands, dass es bereits Literaturanalysen zur CDO-Thematik gibt, kann auf der Basis der hier beispielhaft vorgestellten Artikel konstatiert werden, dass die vorhandenen Literaturanalysen auf theoretische Implikationen und Forschungslücken fokussieren, jedoch weitgehend die Darlegung spezifischer Handlungsempfehlungen für Unternehmen vernachlässigen.

Vor diesem Hintergrund haben wir die einschlägige Fachliteratur zur Rolle des CDOs systematisch analysiert, um darauf aufbauend Handlungsempfehlungen für die Praxis abzuleiten. Bei drei der in diesem Beitrag analysierten Fragen handelt es sich um zentrale Fragen der CDO-Fachliteratur; vgl. z. B. Drechsler (2020) und Walchshofer und Riedl (2017), die Aufgaben und Rollen, Anforderungen als auch die Abgrenzung zum CIO adressieren. Zudem thematisieren wir auch die Gründe für die Einstellung eines CDOs. Dies ist ein wichtiger Aspekt, da aktuell viele Unternehmen darüber zu entscheiden haben, ob sie eine CDO-Position schaffen sollen, und wenn ja, warum (Singh et al. 2020). Der vorliegende Beitrag behandelt somit die folgenden vier Forschungsfragen:

1. Was sind Gründe, einen CDO einzustellen?
2. Was sind Aufgaben und Rollen eines CDOs?
3. Was sind Anforderungen an einen CDO?
4. Wie grenzt sich der CDO vom CIO ab?

Der vorliegende Beitrag ist wie folgt aufgebaut: Abschnitt 2 beschreibt die Methode der Literaturanalyse, Abschnitt 3 präsentiert die Ergebnisse, Abschnitt 4 diskutiert die Ergebnisse und leitet daraus Handlungsempfehlungen ab. Der Beitrag schließt in Abschnitt 5 mit einem Fazit und den Einschränkungen des Erkenntnisanspruchs.

## Methode der Literaturanalyse

Diese Literaturanalyse basiert auf einem Vorgehensmodell für systematische Literaturanalysen nach vom Brocke et al. (2009). Die Literaturrecherche umfasste empirische CDO-Studien sowie konzeptionelle Beiträge zur CDO-Position. Insbesondere wurden die in den Studien verwendeten Forschungsmethoden sowie die Forschungsergebnisse analysiert. Bei den konzeptionellen Beiträgen wurden die Kernaussagen untersucht. Diese Literaturanalyse ist als neutrale Zusammenfassung relevanter Studien und als konzeptioneller Artikel konzipiert und umfasst eine Journal- und Datenbanksuche. Für die Journalsuche wurden entsprechende Outlets (A+, A, B, C und D) aus den Teilbereichen Wirtschaftsinformatik (WI) und Allgemeine Betriebswirtschaftslehre (ABWL) des VHB-JOURQUAL3-Rankings (Verband der Hochschullehrer für Betriebswirtschaft e. V. 2019) in die Suche einbezogen. Diese Teilbereiche wurden auch in vorherigen konzeptionellen Studien zum CDO als bedeutsam identifiziert (z. B. Buchwald und Lorenz 2020). Diese Journalsuche ergab 145 Treffer (ohne Duplikate). Darüber hinaus wurde in folgenden Datenbanken nach Journalbeiträgen und Konferenzartikeln recherchiert: ACM Digital Library, AIS e‑library (AISeL), EBSCOhost (Business Source Complete), IEEE Xplore, Scopus und Web of Science. Diese Datenbanken wurden ausgewählt, da sie in ihrem jeweiligen Themengebiet fundierte Inhalte für die Bearbeitung der gegenständlichen Forschungsfragen enthalten. Entsprechend der Forschungsfrage lauteten die verwendeten Suchbegriffe „Chief Digital Officer“ und „Chief Digital Officers“^2^. Bei der Datenbanksuche wurden die Publikationstitel und Abstracts durchsucht und es wurden 44 Treffer (ohne Duplikate) identifiziert.

**Abb. 1:**
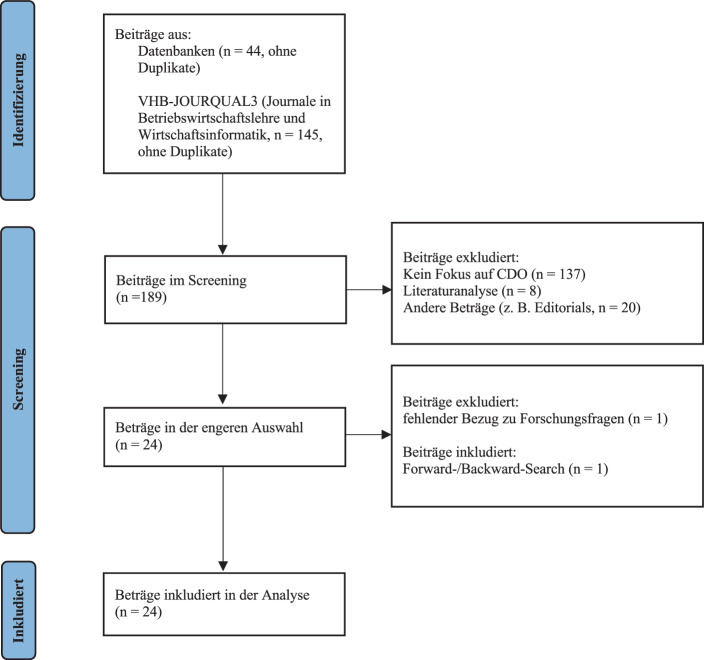
Literatur-Suchprozess. (Adaptierte Version des PRISMA 2020 FLOW CHART nach Page et al. 2021)

Diese initiale Suche ergab somit in Summe 189 Treffer (letzte Suchanfrage: 10. Februar 2022). Nicht einbezogen wurden Beiträge, die sich nicht direkt mit Inhalten zu den Forschungsfragen befassen (sich z. B. mit Strategien zur digitalen Transformation beschäftigen, *n* = 137). Außerdem haben wir *n* = 28 Beträge eliminiert, welche weder als empirische Studien noch als konzeptionelle Artikel klassifiziert werden konnten (*n* = 8 Literaturanalysen und *n* = 20 andere Beitragsarten wie Editorials und Research-in-Progress-Artikel). Nach einer eingehenden Analyse haben wir außerdem Seeher et al. (2020) eliminiert, da der Fokus dieses Artikels auf der Sammlung von Key-Performance-Indicators (KPIs) für die CDO-Tätigkeit liegt und somit nicht explizit auf Aufgaben oder Anforderungen an einen CDO eingegangen wird. Die Suche nach weiteren Artikeln anhand des Pools an identifizierten Artikeln (Forward‑/Backward-Search) ergab einen zusätzlichen Treffer. Aus den Treffern konnten final *n* = 24 relevante Beiträge identifiziert werden. Abb. 1 enthält eine Übersicht über den Suchprozess. Tab. 5, die im Anhang angeführt ist, stellt des Weiteren die identifizierten Artikel anhand von folgenden Kriterien vor: Autor(en) der Studie; Land, in dem die Studie durchgeführt wurde; Methode(n) und Stichprobengröße der Studie. Im nächsten Schritt haben wir die Forschungsergebnisse (bei empirischen Studien) sowie die Kernaussagen (bei konzeptionellen Beträgen) der relevanten Beiträge im Hinblick auf unsere Forschungsfragen analysiert. Bei dieser Analyse wurden durch wiederholtes Lesen Schlüsselwörter und Sätze identifiziert, um daraus die Kernaussagen abzuleiten. Diese wurden dann gruppiert und anhand dieser Gruppierungen wurden Kategorien für jede Forschungsfrage abgeleitet (Webster und Watson 2002). Im nächsten Kapitel diskutieren wir die Ergebnisse unserer Literaturanalyse. Tab. 1, 2 und 3 schaffen darüber hinaus eine Übersicht über die identifizierten Kategorien, Anzahl der Nennungen je Kategorie sowie den korrespondierenden Autoren (Webster und Watson 2002).

**Tab. 1:** Meistgenannte Gründe, warum Unternehmen eine CDO-Position einrichten

Meistgenannte Gründe	Anzahl	Autoren
Strategische Führung bei Veränderungen	7	Haffke et al. (2016); Singh und Hess (2017); Tumbas et al. (2017); Berman et al. (2020); Buchwald und Lorenz (2020); Kunisch et al. (2022); Firk et al. (2021)
CIO und IT-abteilungsbezogene Gründe	4	Haffke et al. (2016); Tumbas et al. (2017); Buchwald und Lorenz (2020); Kunisch et al. (2022)
Hoher Transformations- und Digitalisierungsdruck	4	Haffke et al. (2016); Singh und Hess (2017); Buchwald und Lorenz (2020); Firk et al. (2021)
Marketingbezogene Gründe (z. B. das Optimieren der CX, Erhöhung der Kundenfokussierung)	4	Haffke et al. (2016); Tumbas et al. (2017); Buchwald und Lorenz (2020); Kunisch et al. (2022)
Wertsteigerung des Unternehmens	3	Drechsler et al. (2019); Berman et al. (2020); Kunisch et al. (2022)

**Tab. 2:** Meistgenannte Aufgaben eines CDOs

Meistgenannte Aufgaben	Anzahl	Autoren
Digitalisierung und Implementieren der digitalen Transformation	16	Haffke et al. (2016); Horlacher und Hess (2016); Singh und Hess (2017); Walchshofer und Riedl (2017); Becker et al. (2018); Tahvanainen und Luoma (2018); Ctarino et al. (2018); Doonan (2018); Tumbas et al. (2018); Reck und Fliaster (2019); Wade und Obwegeser (2019); Artemenko (2020); Berman et al. (2020); Buchwald und Lorenz (2020); Kunisch et al. (2022); Danilova et al. (2022)
Programm- und Projekt-Management, Produktentwicklung	15	Haffke et al. (2016); Horlacher und Hess (2016); Tumbas et al. (2017, 2018); Walchshofer und Riedl (2017); Singh und Hess (2017); Becker et al. (2018); Tahvanainen und Luoma (2018); Doonan (2018); Suheimat et al. (2018); Wade und Obwegeser (2019); Reck und Fliaster (2019); Artemenko (2020); Buchwald und Lorenz (2020); Danilova et al. (2022)
Neue Technologien identifizieren und einführen	11	Haffke et al. (2016); Walchshofer und Riedl (2017); Singh und Hess (2017); Tumbas et al. (2017); Doonan (2018); Tahvanainen und Luoma (2018); Wade und Obwegeser (2019); Artemenko (2020); Buchwald und Lorenz (2020); Kunisch et al. (2022); Danilova et al. (2022)
Optimierung des Marketings (zum Beispiel der Kundenerfahrung)	10	Haffke et al. (2016); Horlacher und Hess (2016); Tumbas et al. (2017, 2018); Tahvanainen und Luoma (2018); Wade und Obwegeser (2019); Becker und Jaakkola (2020); Buchwald und Lorenz (2020); Kunisch et al. (2022); Danilova et al. (2022)
Unternehmenskultur ändern	10	Haffke et al. (2016); Singh und Hess (2017); Walchshofer und Riedl (2017); Becker et al. (2018); Ctarino et al. (2018); Tahvanainen und Luoma (2018); Reck und Fliaster (2019); Berman et al. (2020); Buchwald und Lorenz (2020); Danilova et al. (2022)
Unternehmens- und Digitalstrategie entwickeln	8	Haffke et al. (2016); Walchshofer und Riedl (2017); Ctarino et al. (2018); Wade und Obwegeser (2019); Berman et al. (2020); Buchwald und Lorenz (2020); Singh et al. (2020); Danilova et al. (2022)
Vorantreiben von Veränderungen, Changemanagement	6	Ctarino et al. (2018); Suheimat et al. (2018); Tahvanainen und Luoma (2018); Buchwald und Lorenz (2020); Singh et al. (2020); Danilova et al. (2022)
Mitarbeiter motivieren	6	Walchshofer und Riedl (2017); Suheimat et al. (2018); Tahvanainen und Luoma (2018); Reck und Fliaster (2019); Artemenko (2020); Danilova et al. (2022)
Mitarbeiter weiterbilden	6	Haffke et al. (2016); Horlacher und Hess (2016); Walchshofer und Riedl (2017); Tahvanainen und Luoma (2018); Reck und Fliaster (2019); Danilova et al. (2022)
Vorantreiben von Innovationen	5	Reck und Fliaster (2019); Buchwald und Lorenz (2020); Singh et al. (2020); Kunisch et al. (2022); Danilova et al. (2022)
Etablieren von Kollaboration (z. B. mit Start-Ups), internes und externes Netzwerken	5	Walchshofer und Riedl (2017); Tumbas et al. (2018); Reck und Fliaster (2019); Buchwald und Lorenz (2020); Danilova et al. (2022)
Prozesse optimieren	4	Horlacher und Hess (2016); Walchshofer und Riedl (2017); Artemenko (2020); Danilova et al. (2022)
Kommunikation/Fürsprecher für die IT bzw. die digitale Transformation	4	Haffke et al. (2016); Horlacher und Hess (2016); Buchwald und Lorenz (2020); Danilova et al. (2022)

**Tab. 3:** Meistgenannte Anforderungen, die ein CDO benötigt

Meistgenannte Anforderungen	Anzahl	Autoren
Strategisches und geschäftsorientiertes Denken	11	Horlacher und Hess (2016); Walchshofer und Riedl (2017); Tumbas et al. (2017); Ctarino et al. (2018); Tahvanainen und Luoma (2018); Reck und Fliaster (2019); Wade und Obwegeser (2019); Artemenko (2020); Berman et al. (2020); Buchwald und Lorenz (2020); Singh et al. (2020)
Sozialkompetenz und Kommunikationsfähigkeiten	10	Horlacher und Hess (2016); Tumbas et al. (2017); Walchshofer und Riedl (2017); Ctarino et al. (2018); Doonan (2018); Tahvanainen und Luoma (2018); Reck und Fliaster (2019); Wade und Obwegeser (2019); Buchwald und Lorenz (2020); Singh et al. (2020)
Technologie/IT Know-how	10	Horlacher und Hess (2016); Singh und Hess (2017); Walchshofer und Riedl (2017); Tahvanainen und Luoma (2018); Reck und Fliaster (2019); Wade und Obwegeser (2019); Artemenko (2020); Berman et al. (2020); Buchwald und Lorenz (2020); Singh et al. (2020)
Changemanagement	7	Horlacher und Hess (2016); Singh und Hess (2017); Tumbas et al. (2017); Walchshofer und Riedl (2017); Doonan (2018); Tahvanainen und Luoma (2018); Buchwald und Lorenz (2020)
Kundenorientierung	6	Tumbas et al. (2017); Doonan (2018); Tahvanainen und Luoma (2018); Wade und Obwegeser (2019); Artemenko (2020); Buchwald und Lorenz (2020)
Analytische Fähigkeiten	4	Tumbas et al. (2017); Tahvanainen und Luoma (2018); Artemenko (2020); Buchwald und Lorenz (2020)
Erfahrungen in der digitalen Transformation und Digitalisierung	4	Tumbas et al. (2017); Walchshofer und Riedl (2017); Tahvanainen und Luoma (2018); Wade und Obwegeser (2019)
Innovationsstreben	4	Tumbas et al. (2017); Reck und Fliaster (2019); Berman et al. (2020); Buchwald und Lorenz (2020)
Führungsqualitäten	3	Walchshofer und Riedl (2017); Ctarino et al. (2018); Tahvanainen und Luoma (2018)
Visionäres Denken	2	Ctarino et al. (2018); Tahvanainen und Luoma (2018)
Problemlösungsorientierung	2	Ctarino et al. (2018); Tahvanainen und Luoma (2018)
Inspirierende Ausstrahlung	2	Singh und Hess (2017); Tahvanainen und Luoma (2018)

## Ergebnisse

### Was sind Gründe, einen CDO einzustellen?

Von den 24 Artikeln behandeln 8 Artikel mögliche unternehmensbezogene Gründe für das Einrichten bzw. Vorhandenseins eines CDOs. Mit 7 Nennungen war der Bedarf der *strategischen Führung bei Veränderungen *der am meisten genannte Grund, warum Unternehmen eine CDO-Position schaffen bzw. geschaffen haben. Die Autoren wiesen in diesem Kontext darauf hin, dass der CDO Veränderungen zentral steuern sollte und die Veränderungen auch kommunizieren sollte (Haffke et al. 2016; Firk et al. 2021). Ob es nötig ist, Veränderungen zentral zu steuern, hängt auch von der Unternehmenskultur und der internen Bereitschaft zur Kollaboration ab (Haffke et al. 2016; Singh und Hess 2017). Ein weiterer Einflussfaktor war die Firmengröße (z. B. Haffke et al. 2016; Buchwald und Lorenz 2020). So zeigte sich ein positiver Zusammenhang zwischen der Firmengröße und dem Vorhandensein eines CDOs, was darauf hindeutet, dass bei größeren Firmen der Bedarf nach einer zentralen Steuerung größer ist (Kunisch et al. 2022). Ebenfalls für eine zentrale Steuerung und Koordination der Aktivitäten zur digitalen Transformation spricht eine hohe interne Komplexität der Tätigkeiten (z. B. wenn es bislang keine unternehmensweite Abstimmung der einzelnen Aktivitäten zur digitalen Transformation gibt, Singh und Hess 2017) und der Umstand einer fehlenden unternehmensweiten Digitalstrategie (Tumbas et al. 2017).

Des Weiteren stellen 4 Artikel *marketingbezogene Gründe* für das Einstellen eines CDO vor. So korrelierte die Marketingintensität positiv mit dem Vorhandensein eines CDOs (Kunisch et al. 2022). Durch das hohe Aufkommen von Kundendaten (z. B. durch Kundenkarten oder soziale Medien) und die immer komplexer werdende Customer Journey wird auch im Marketing immer mehr technologisches Know-how benötigt (Tumbas et al. 2017). Der CDO kann somit insbesondere auch als Schnittstelle zwischen Marketing und IT-Abteilung benötigt werden (Tumbas et al. 2018). Auch Unternehmen, bei welchen die Digitalisierung vergleichsweise stark von außen wahrgenommen werden kann (z. B. Unternehmen im Bereich Sales oder Marketing), haben oft einen größeren Bedarf nach einem CDO, als Unternehmen die tendenziell in intern-fokussierten Unternehmensbereichen tätig sind (z. B. Unternehmen der Logistik, Haffke et al. 2016; Buchwald und Lorenz 2020).

Vier Artikel sehen *keine existierende CIO-Position bzw. eine IT-Abteilung oder einen CIO, der nicht kundenfokussiert arbeitet,* als Grund, eine CDO-Position zu schaffen. In manchen Unternehmen übernimmt der CIO die Aufgaben des CDOs (z. B. Horlacher und Hess 2016). Sofern dies allerdings nicht der Fall ist, wenn es beispielsweise keinen CIO gibt oder der CIO sich rein auf die technischen Belange beschränkt, kann es sich als zweckmäßig erweisen, einen CDO einzustellen (Tumbas et al. 2017). Die Umsetzung von Projekten der digitalen Transformation benötigt auch einen gewissen Zuspruch im Unternehmen. Sollte der CIO daher einen schlechten Ruf im Unternehmen haben, könnte die Einstellung eines CDOs ebenfalls zweckmäßig sein (Haffke et al. 2016). Darüber hinaus sehen 4 Artikel die Notwendigkeit für einen CDO, wenn das Unternehmen einem *hohen Transformations- und Digitalisierungsdruck* unterliegt. Buchwald und Lorenz (2020) halten zum Beispiel in diesem Zusammenhang fest, „[t]he pressure for digitization mostly relates to the external environment involving changes in customer needs, competitor advances, and new disruptive market entrants“ (S. 10). Insgesamt 3 Artikel sahen außerdem die *Wertsteigerung des Unternehmens* als Grund für die Etablierung eines CDOs. Drechsler et al. (2019) haben herausgefunden, dass die Einstellung eines spezialisierten und auf die individuelle Digitalstrategie zugeschnittenen CDOs den Aktienwert eines Unternehmens steigern kann. Tab. 1 gibt einen zusammenfassenden Überblick über Gründe, warum Unternehmen eine CDO-Position einrichten (ein Grund musste in der Fachliteratur zumindest zwei Mal genannt sein, um Eingang in die Aufstellung zu finden).

### Was sind Aufgaben und Rollen eines CDOs?

Von den 24 analysierten Artikeln diskutieren 19 Beiträge mögliche Aufgaben von CDOs. Die Ergebnisse zeigen, dass mit 16 Nennungen die *Digitalisierung *bzw. das* Implementieren der digitalen Transformation* die meistgenannte Aufgabe eines CDOs ist. Der CDO sollte somit sicherstellen, dass die Digitalstrategie des Unternehmens umgesetzt wird (z. B. Becker et al. 2018). Dieses kann er zum Beispiel dadurch gewährleisten, dass er dazu beiträgt, neue Geschäftsmodelle zu entwickeln sowie neue Einnahmequellen via E‑Commerce zu etablieren (z. B. Becker et al. 2018). Die am zweithäufigsten genannte Aufgabe ist das *Programm- und Projekt-Management* inklusive der Produktentwicklung (15 Artikel). Daraus folgt, dass auch die Projektkoordination und -überwachung im Zusammenhang mit Digitalisierungsinitiativen eine Zuständigkeit des CDOs ist (siehe hierzu auch Riedl 2019). An dritter Stelle wird die *Identifizierung und Implementierung von neuen Technologien *genannt (11 Nennungen). Hierzu zählen beispielsweise das Überwachen von Markttrends im Bereich neuer Technologien sowie das Implementieren von technologischen Innovationen (z. B. Danilova et al. 2022).

Die Optimierung des Marketings (optimieren des Customer Relationship Management, der CX und der Customer Journey) wurde mit 10 Nennungen ebenfalls als Aufgabe des CDOs gesehen. Somit ist der CDO ein wichtiger Akteur, um sicherzustellen, dass bei jeglichen Angeboten des Unternehmens – zum Beispiel technologische Angebote wie die eigene App oder Website – das benutzerfreundliche Design beachtet und optimal in die CX integriert wird (Berman et al. 2020). Ebenfalls mit 10 Nennungen folgt das *Verändern der Unternehmenskultur*. In diesem Bereich sind vor allem die sozialen Fähigkeiten des CDOs gefragt, um beispielsweise die Akzeptanz zur Durchführung von Maßnahmen zur digitalen Transformation im Unternehmen zu steigern (z. B. Danilova et al. 2022).

Obwohl strategisches Denken in den von uns analysierten Artikeln die am meisten genannte Anforderung an den CDO war (siehe Kapitel 3.3.), sahen das *Entwickeln der Unternehmens- und Digitalstrategie* lediglich 8 Artikel als Aufgabe des CDO. Der CDO arbeitet auch aktiv mit den Mitarbeitern (*Vorantreiben von Veränderungen/Changemanagement*, 6 Nennungen; *Weiterbilden von Mitarbeitern*, 6 Nennungen; *Mitarbeitermotivation*, 6 Nennungen). Das *Etablieren von Kollaborationen* intern, aber auch extern (z. B. mit Kunden oder Start-ups) und das *generelle Innovationsmanagement* (5 Nennungen) sowie das *Optimieren von Prozessen* und die *Kommunikation bzw. ein Fürsprecher für die IT bzw. die digitale Transformation sein* (4 Nennungen) wurden weniger oft genannt. Tab. 2 gibt einen Überblick über die Aufgaben eines CDOs, die mindestens zweimal in der Fachliteratur genannt wurden.

### Was sind Anforderungen an einen CDO?

Von den 24 analysierten Artikeln nennen 13 Artikel Anforderungen an den CDO. Mit 11 Nennungen ist das *strategische bzw. das geschäftsorientierte Denken* die am meisten genannte Anforderung. Diese häufige Nennung stimmt auch mit den am häufigsten genannten Aufgaben eines CDOs überein, laut welchen er dazu beiträgt, die Maßnahmen zur digitalen Transformation zu implementieren und neue Geschäftsmodelle sowie die Digitalstrategie zu entwickeln (siehe Tab. 2). Neben geschäftsorientiertem Denken sollte der CDO auch *Technologie- und IT-Know-how* mitbringen (Nennung in insgesamt 10 Artikeln). Des Weiteren wurden *soziale Kompetenzen* (zwischenmenschliche Fähigkeiten wie Einfühlungsvermögen oder Team‑, Kommunikations-, und Kollaborationsfähigkeiten) in 10 Artikeln genannt. Auch *Erfahrungen im Changemanagement* (7 Artikel) und eine *Kundenorientierung* (6 Artikel) im Bereich CX und User Experience Management sind genannte Anforderungen. Sowohl *analytische Fähigkeiten, vorherige Erfahrungen in der digitalen Transformation* und *Innovationsfähigkeiten* (jeweils 4 Nennungen) als auch *Führungsqualität *(3 Nennungen),* visionäres Denken, Problemlösungsorientierung* und eine *inspirierende Ausstrahlung *(jeweils 2 Nennungen) gehören zu den weniger oft genannten Anforderungen. Zusammenfassend bleibt auch festzuhalten, dass zwar viele der hier genannten Anforderungen erlernbar sind (z. B. Technologie-Know-how), manche Anforderungen allerdings nur schwer erlernbar sind (z. B. eine inspirierende Ausstrahlung). Tab. 3 gibt einen Überblick über die Anforderungen, die an einen CDO gestellt werden (es werden Anforderungen gelistet, die mindestens zweimal in der Fachliteratur genannt wurden).

Walchshofer und Riedl (2017) halten darüber hinaus fest, dass alle Interviewpartner in ihrer Untersuchung ein Hochschulstudium oder eine entsprechende Ausbildung im geforderten Umfeld abgeschlossen haben. Zhan et al. (2020) analysierten den Bildungshintergrund von 112 neu berufenen CDOs. Von den eingestellten CDOs hatten 29 % einen Bachelorabschluss, 43 % einen Masterabschluss, 25 % einen Ph.D. und 33 % einen MBA (Master of Business Administration). In einer Studie von Berman et al. (2020) gaben 84 % der Befragten an, einen STEM-Bachelorabschluss (science, technology, engineering, mathematics) zu haben. Weitere 62 % der Befragten haben einen über den Bachelor hinausgehenden Abschluss im Bereich Business oder Management. Eine weitere Studie (Drechsler et al. 2019) hat sich damit beschäftigt, wie sich der Aktienwert eines Unternehmens als Konsequenz auf die Einstellung eines CDO mit oder ohne STEM-Hintergrund verändert. Die Analyse zeigt, dass sich der Aktienwert positiv verändert, wenn der CDO keinen STEM-Hintergrund hat, sich jedoch negativ verändert, wenn der CDO einen STEM-Hintergrund hat. Dieses Ergebnis unterstreicht, dass insbesondere Shareholdern wichtig zu sein scheint, dass der CDO keinen rein technisch-mathematischen Hintergrund hat.

### Wie grenzt sich der CDO vom CIO ab?

Die digitale Transformation reicht auch in den Zuständigkeitsbereich des CIOs und in der Fachliteratur wird daher immer wieder diskutiert, wie sich die Zuständigkeiten eines CDOs von denen eines CIOs abgrenzen lassen (z. B. Walchshofer und Riedl 2017). Im Folgenden gehen wir daher auf jene Artikel ein, die Profile, Aufgaben und die Ausrichtung der CDO- bzw. der CIO-Position thematisieren. Des Weiteren diskutieren wir die mögliche Zusammenarbeit zwischen CDO und CIO mit anderen C‑Level-Managern.

#### CDO vs. CIO: Ausrichtung, Profil und Aufgaben

Walchshofer und Riedl (2017) halten fest, dass die Aufgaben eines CDOs und eines CIOs zum Teil ähnlich sind (zum Beispiel bei zukunftsweisenden Überlegungen und Ausführungen im Rahmen der Visions- bzw. Strategieentwicklung oder Aufgaben im IT-Controlling). Bei manchen Aufgaben hat jedoch der CDO ein weiteres Aufgabenspektrum als der CIO. Zum Beispiel zählt das Überwachen von neuen Trends und Technologien zum Aufgabenbereich von CDO und CIO. Der CDO soll allerdings über das reine Überwachen von Trends hinausgehend Innovationen schaffen und disruptive Geschäftsmodelle entwickeln. Walchshofer und Riedl (2017) halten auch fest, dass der CIO auf die eigenen Mitarbeiter fokussiert ist, wohingegen der CDO sowohl die internen als auch die externen Prozesse verbessert (alle Mitarbeiter sowie Kunden sind im Fokus). Daher hat insbesondere der CDO weitere Aufgaben im Bereich Kommunikation, Netzwerken und Repräsentation nach außen sowie Verändern der Unternehmenskultur. Die Autoren fassen zusammen: „Als Resümee kann die Arbeit des CDOs im Vergleich zu jener des CIOs als übergeordnet (im Sinne von strategischer ausgerichtet) bezeichnet werden. Die Person ist funktionsübergreifend tätig und die Digitalisierung bzw. die digitale Transformation des Unternehmens stehen im Fokus“ (Walchshofer und Riedl, 2017, S. 334).

Diese grundlegende Aufteilung der Zuständigkeiten eines CIOs als Führungskraft mit Kernaufgaben im IT-Bereich und eines CDOs als Führungskraft im Bereich Innovation, Kundenorientierung sowie der Strategie und Kommunikation der digitalen Transformation teilen verschiedene Wissenschaftler (Haffke et al. 2016; Horlacher und Hess 2016; Singh und Hess 2017; Tumbas et al. 2017; Ctarino et al. 2018; Tahvanainen und Luoma 2018; Buchwald und Lorenz 2020). Ctarino et al. (2018) halten beispielsweise fest, dass das Profil eines CDOs geschäftsorientiert, aber auch visionär ist und, dass der CDO Führungsqualitäten besitzen sollte. Des Weiteren benötigen CDOs eine höhere Risikobereitschaft, strategisches Denken und Kompetenzen im interpersonellen Beziehungsaufbau (Ctarino et al. 2018). Der CIO hingegen hat laut Ctarino et al. (2018) insbesondere ein ausgeprägtes IT-Verständnis, ist detailorientiert, ergebnisorientiert und seine Fähigkeiten liegen vor allem in der technischen Führung.

Aus dieser strikten Aufgabenverteilung ergibt sich, dass sich die Rollen von CDO und CIO zwar unterscheiden, die Positionen aber auch voneinander abhängen (z. B. Horlacher und Hess 2016). Der CDO ist somit eine Art Fürsprecher für die IT und die Einführung einer CDO-Position birgt somit die Chance auf mehr Sichtbarkeit der IT (Haffke et al. 2016; Singh und Hess 2017; Buchwald und Lorenz 2020). CIOs sollten daher durch eine enge Zusammenarbeit mit dem CDO die Chance auf eine höhere Sichtbarkeit nutzen (Singh und Hess 2017). Auch Tumbas et al. (2017) stimmen zu, dass beide Rollen als komplementär anzusehen sind und, dass der CDO manchmal als Mittler zwischen dem Unternehmen und der IT fungiert. Buchwald und Lorenz (2020) halten außerdem fest, dass für eine gute Beziehung zwischen CDO und CIO beide Positionen auf derselben Hierarchiestufe angesiedelt sein sollten und dass ein gemeinsames Verständnis der jeweiligen Rollen, Aufgaben und individuellen Sach- und Fachkenntnissen wichtig ist. Zhan und Mu (2016) analysierten, welche Auswirkungen mögliche Job-Überlappungen von CIOs und CDOs auf den Aktienwert eines Unternehmens haben. Ihre Analyse zeigt, dass bei der Neueinstellung eines CDOs, welcher Überlappungen mit den Zuständigkeiten des CIOs hat, eine negative Auswirkung auf den Aktienkurs des Unternehmens zu beobachten ist. Weitere Studien von Zhan et al. (2020) und Drechsler et al. (2019) bestätigen, dass sich die Ankündigung einer CDO-Einstellung positiv auf den Aktienwert auswirkt, wenn Firmen keinen CIO haben. Die Ergebnisse dieser Studien unterstreichen die Wichtigkeit für Unternehmen, bereits vor der Einstellung mögliche Konflikte zwischen CIO und CDO zu antizipieren und verhindern (z. B. durch das genaue Festlegen von Zuständigkeiten für CIO und CDO, Kunisch et al. 2022).

Darüber hinaus muss das Unternehmen festlegen, wie viele und wie die CDO-Position(en) geschaffen werden sollen. Sowohl Singh et al. (2020) als auch Horlacher et al. (2016) diskutieren in diesem Zusammenhang eine zentrale CDO-Position (das Unternehmen hat einen CDO) vs. eine dezentrale CDO-Ausrichtung (unterschiedliche Unternehmensbereiche haben jeweils einen CDO). Berman et al. (2020) fanden in ihrer Umfrage heraus, dass der überwiegende Teil (65 %) der befragten CDOs für die Digitalisierung im Unternehmen verantwortlich ist. Weitere 29 % teilen sich die Verantwortung mit einem Team oder anderen Führungskräften und nur 6 % der Befragten haben keine direkten Verantwortlichkeiten im Rahmen der digitalen Transformation sondern fungieren rein als Influencer. Im Sinne der formalen Einbindung kann der CDO zum Beispiel in das Top-Managementteam aufgenommen werden oder rein informal agieren, zum Beispiel durch das Anbieten von freiwilligen Mitarbeiter-Workshops (Horlacher et al. 2016; Singh et al. 2020). Betrachtet man also die möglichen Ausrichtungen der CDO-Position, so wird klar, dass auch diese einen Effekt darauf haben, wie der CDO mit dem CIO und anderen C‑Level-Managern kommuniziert und zusammenarbeitet.

Eine weitere Option für Unternehmen ist, die CDO- und CIO-Rollen in einer Person zu vereinen. Dieses Modell beruht auf der Diskussion, ob es nötig ist, neben dem CIO noch einen CDO einzustellen (z. B. Mani 2017) oder ob der CIO auch die Belange eines CDOs übernehmen kann. Tumbas et al. (2017) geben zum Beispiel konkrete Gründe an, warum ein CDO eingestellt werden sollte. Sie schlagen vor, dass ein CDO eingestellt werden sollte, wenn die IT-Abteilung mit der IT-Infrastruktur in einem Maße beschäftigt ist, dass keine oder wenig Kapazitäten für die digitale Transformation vorhanden sind.

#### CDO, CIO und andere C-Level-Manager

Wissenschaftler beschäftigten sich ebenfalls mit der Frage, wie der CDO mit dem CIO, aber auch mit anderen C‑Level-Managern zusammenarbeiten kann. Tumbas et al. (2018) schlagen drei unterschiedliche Vorgehensweisen zwischen bereits vorhandenen Abteilungen und der Digitalisierungsabteilung vor. Bei dem sogenannten Grafting besteht eine enge Verknüpfung zwischen dem CDO und den bestehenden Funktionseinheiten. Die Funktionseinheiten werden von der Digitalabteilung unterstützt. Der CDO arbeitet somit mit vielen unterschiedlichen Funktionen wie der IT-Abteilung, den Fachabteilungen sowie der Geschäftsführung zusammen. Beim sogenannten Bridging fungiert der CDO als Brücke zwischen zwei Funktionseinheiten (zum Beispiel Marketing und IT) und seine Rolle ist hier zeitlich begrenzt. Beim Decoupling werden die digitalen Initiativen isoliert von bereits existierenden Funktionseinheiten gehandhabt. Tumbas et al. (2018) weisen aber auch darauf hin, dass es insbesondere beim Decoupling schwierig ist, die digitale Transformation im ganzen Unternehmen zu implementieren (insbesondere, weil die bereits vorhandene IT-Abteilung im Prozess nicht mit einbezogen wird).

Walchshofer und Riedl (2017) schlagen vor, dass der CDO direkt dem CEO unterstellt sein sollte. Eine Studie von Berman et al. (2020) zeigt jedoch auch, dass bei Unternehmen, die ihre Wettbewerber übertreffen (Outperformer), der CDO direkt dem CIO berichtet. Buchwald und Lorenz (2020) leiten ab, dass ein Vorteil, den CIO in das Top-Management aufzunehmen, darin liegt, dass IT als wichtig für strategische Firmenentscheidungen gesehen wird und die IT-Strategie mit der Unternehmensstrategie überein gebracht werden kann. Ein Aufnehmen des CIOs in das Top-Management kann somit ebenfalls positive Auswirkungen auf die Unternehmensleistung haben und eine regelmäßige Kommunikation zwischen dem CDO, CIO, CEO und anderen C‑Level-Managern kann wichtige Netzwerke im Unternehmen bilden und fördern (Buchwald und Lorenz 2020). Insbesondere der CDO kann weitgehende Freiheiten im Unternehmen genießen (z. B. im Zusammenhang mit der Festlegung der Digitalstrategie), die Unterstützung durch und eine Koordination mit dem Top-Management ist jedoch auch für ihn essenziell (Buchwald und Lorenz 2020). Zusammenfassend kann daher gesagt werden, dass eine enge Zusammenarbeit zwischen CIO und CDO eine wichtige Determinante der erfolgreichen digitalen Transformation ist, die Ausgestaltung dieser Zusammenarbeit aber von verschiedenen Gegebenheiten im Unternehmen abhängt (z. B. ob der CDO dem CEO oder dem CIO berichtet, siehe Haffke et al. 2016; Singh und Hess 2017).

## Diskussion der Ergebnisse und Handlungsempfehlungen

Dieser Abschnitt diskutiert die Ergebnisse der Literaturanalyse und leitet Handlungsempfehlungen für die Praxis ab.

Am Beispiel des veränderten Kundenverhaltens (z. B. hin zur Nutzung von Online-Services) wird deutlich, wie wichtig es ist, dass Unternehmen ihre Geschäftsmodelle digitalisieren und die digitale Transformation weiter vorantreiben (z. B. Vial 2019; Verhoef et al. 2021). Fitzgerald et al. (2013) halten in diesem Zusammenhang zum Beispiel fest, dass die digitale Transformation auch „[t]he use of new digital technologies (social media, mobile, analytics or embedded devices) to enable major business improvements (such as enhancing customer experience, streamlining operations or creating new business models)“ (S. 2) beinhaltet. CDOs sind somit ebenfalls dafür verantwortlich, auf Veränderungen im Kundenverhalten zu reagieren und neue Möglichkeiten durch digitale Innovationen und neue Technologien für Unternehmen nutzbar zu machen (z. B. Horlacher und Hess 2016). Unternehmen, die die Chancen von neuen Technologien nutzen und diese Technologien gut in das Unternehmen und seine Geschäftsmodelle integrieren, haben dabei oft höhere Umsätze als ihre Wettbewerber (z. B.. Fitzgerald et al. 2013). Unternehmen, die jedoch nicht im Zeitalter der Digitalisierung mithalten können – zum Beispiel auch, weil Veränderungen nicht auf einem strategischen Level eingeführt werden (Verhoef et al. 2021) oder weil kein CDO vorhanden ist und der CIO bzw. die IT-Abteilung lediglich mit der IT-Infrastruktur und nicht mit der digitalen Transformation vertraut sind (z. B. Tumbas et al. 2017) – riskieren, dass ihre Geschäftsmodelle scheitern (z. B. Hess et al. 2016; Verhoef et al. 2021). Die Digitalisierung und das dadurch veränderte Kundenverhalten haben somit weitreichende Folgen und eine Herausforderung des CDOs ist mit den Wettbewerbern Schritt zu halten (Horlacher und Hess 2016). Zusammenfassend und basierend auf den Ergebnissen der vorliegenden Literaturanalyse definieren wir somit die folgenden Bedingungen, die die Zweckmäßigkeit einer CDO-Position im Unternehmen begründen: (1) Notwendigkeit der strategischen Führung bei Veränderungen, (2) der CIO bzw. die IT-Abteilung arbeiten nicht bereits an der Digitalisierung und der Implementierung der digitalen Transformation, (3) hoher externer Transformations- und Digitalisierungsdruck, (4) Notwendigkeit zur Optimierung des Marketings und der CX sowie (5) Unternehmenswertverluste durch verpasste digitale Transformation (siehe Tab. 4, Forschungsfrage 1).

**Tab. 4:** Übersicht der Handlungsempfehlungen

Forschungsfrage	Handlungsempfehlungen
(1) Was sind Gründe, einen CDO einzustellen?	*Beim Vorliegen folgender Bedingungen ist es zweckmäßig, eine CDO-Position im Unternehmen einzurichten:* – Notwendigkeit der strategischen Führung bei Veränderungen – CIO bzw. IT-Abteilung arbeiten nicht bereits an der strategischen und operativen Implementierung der digitalen Transformation – Hoher externer Transformations- und Digitalisierungsdruck – Notwendigkeit zur Optimierung des Marketings und der CX – Unternehmenswertverluste durch verpasste digitale Transformation
(2) Was sind Aufgaben und Rollen eines CDO?	*Sofern man in einem Unternehmen die CDO-Position einrichtet, sollte der CDO die folgenden Aufgaben übernehmen:* – Digitalisierung und Implementierung der digitalen Transformation – Programm- und Projekt-Management, Produktentwicklung – Identifizierung und Einführung neuer Technologien – Optimierung des Marketings – Änderung der Unternehmenskultur hin zu einer größeren Akzeptanz der Aktivitäten zur digitalen Transformation – Entwicklung der Unternehmens- und Digitalstrategie – Vorantreiben von Veränderungen/Changemanagement – Motivation von Mitarbeitern – Weiterbildung von Mitarbeitern – Innovationsmanagement – Etablieren von internen und externen Netzwerken und Kollaborationen – Optimierung von Prozessen – Fungieren als Fürsprecher für die digitale Transformation durch gezielte Kommunikation
(3) Was sind Anforderungen an einen CDO?	*Unternehmen, die einen CDO einstellen wollen, sollten auf folgende Anforderungen bei potenziellen Kandidaten achten:* – Strategisches und geschäftsorientiertes Denken – Sozialkompetenz und Kommunikationsfähigkeiten – Technologie/IT Know-how – Erfahrungen im Changemanagement – Kundenorientierung – Analytische Fähigkeiten – Erfahrung in der digitalen Transformation und Digitalisierung – Innovationsstreben – Führungsqualitäten – Visionäres Denken – Problemlösungsorientierung – Inspirierende Ausstrahlung
(4) Wie grenzt sich der CDO vom CIO ab?	*Unternehmen, die sowohl einen CDO als auch einen CIO beschäftigen, sollten auf folgendes achten:* – Ausrichtung, Profil und Aufgaben: Der CDO wird primär als Stratege gesehen, der CIO hingegen als Verantwortlicher für die IT-Infrastruktur – CDO, CIO und andere C‑Level-Manager: Sowohl der CIO als auch der CDO sollten gut miteinander – aber auch mit anderen C‑Level-Managern – zusammenarbeiten; die dafür notwendigen Bedingungen sind von der Organisation zu schaffen (z. B. Kommunikationsstrukturen). Der CDO ist dabei meist dem CEO unterstellt. Es sollten KPIs festgelegt werden, damit eine Priorisierung der Projekte zur digitalen Transformation sichergestellt ist

Wenn sich ein Unternehmen entschieden hat, eine CDO-Position einzurichten, sollte der CDO – basierend auf den Ergebnissen der vorliegenden Literaturanalyse – mit folgenden Aufgaben betraut werden: (1) Digitalisierung und Implementierung der digitalen Transformation, (2) Programm- und Projekt-Management sowie Produktentwicklung, (3) Identifizierung und Einführung neuer Technologien, (4) Optimierung des Marketings, (5) Änderung der Unternehmenskultur hin zu einer größeren Akzeptanz der Aktivitäten zur digitalen Transformation, (6) Entwicklung der Unternehmens- und Digitalstrategie, (7) Vorantreiben von Veränderungen/Changemanagement, (8) Motivation von Mitarbeitern, (9) Weiterbildung von Mitarbeitern, (10) Innovationsmanagement, (11) Etablieren von internen und externen Netzwerken und Kollaborationen, (12) Optimierung von Prozessen und (13) Fungieren als Fürsprecher für die digitale Transformation durch gezielte Kommunikation (siehe Tab. 4, Forschungsfrage 2). Unternehmen können des Weiteren auch unterschiedliche Schwerpunkt für die CDO-Position festlegen. Beispielhaft kann der CDO als Evangelist (Fürsprecher für das Implementieren und Nutzen von neuen Technologien) sowie als Innovator, welcher neue Technologien identifiziert und einführt, eingestellt werden (z. B. Buchwald und Lorenz 2020). Dieses weite Aufgabenspektrum veranschaulicht auch, dass die unternehmensinterne Weiterentwicklung hin zu einem höheren Digitalisierungsgrad nicht nur in einem Unternehmensbereich wie zum Beispiel der IT-Abteilung angesiedelt ist (z. B. Horlacher und Hess 2016).

Die Umsetzung der digitalen Transformation ist somit oft mit tiefgreifenden organisatorischen Veränderungen samt der Überarbeitung aktueller Geschäftsmodelle verbunden (z. B. Verhoef et al. 2021). Dies kann anhand des Beispiels des veränderten Kundenverhaltens transparent gemacht werden. Heutzutage stehen dem Unternehmen eine Vielzahl von zusätzlichen Kundeninformationen zur Verfügung, und Unternehmen können diese Daten unter anderem zur Optimierung des Marketings und der CX nutzen (Wedel und Kannan 2016; Lemon und Verhoef 2016; Verhoef et al. 2021). Da die individuelle CX ebenfalls durch viele persönliche und psychologische Faktoren bestimmt wird, gewinnt das Kennen des eigenen Kunden und seiner Wünsche zusätzlich an Bedeutung (z. B. Gentile et al. 2007; Lemon und Verhoef 2016; Hermes und Riedl 2020, 2021a, b). Die integrierte Nutzung der Unternehmensdaten sowohl aus dem Bereich des Marketings als auch aus der IT-Abteilung sind daher essentiell, um die eigenen Geschäftsmodelle anzupassen (z. B. Hirsh et al. 2012; Eckles et al. 2018; Lim et al. 2021). Unternehmen sollten die CDO-Position daher so ausrichten, dass der CDO sowohl als Mittler zwischen dem Unternehmen und den Kunden, aber auch zwischen unterschiedlichen Abteilungen wie der Marketing- und IT-Abteilung agieren kann (z. B. Horlacher und Hess 2016; Berman et al. 2020).

Um dieser anspruchsvollen CDO-Aufgabe gerecht zu werden, sollten Unternehmen daher bei CDO-Kandidaten auf das Vorhandensein von folgenden Anforderungen – basierend auf den Ergebnissen unserer Literaturanalyse – achten: (1) strategisches und geschäftsorientiertes Denken, (2) Sozialkompetenz und Kommunikationsfähigkeiten, (3) Technologie/IT Know-how, (4) Erfahrung im Changemanagement, (5) Kundenorientierung, (6) analytische Fähigkeiten, (7) Erfahrungen in der digitalen Transformation und Digitalisierung, (8) Innovationsstreben, (9) Führungsqualitäten, (10) visionäres Denken, (11) Problemlösungsorientierung und (12) eine inspirierende Ausstrahlung (siehe Tab. 4, Forschungsfrage 3). Unternehmen müssen daher ebenfalls interne Strukturen schaffen, die es dem CDO erlauben, übergreifend zu agieren (Buchwald und Lorenz 2020), Akzeptanz von Veränderungen zu schaffen (z. B. Haffke et al. 2016) und Mitarbeiter und Schlüsselakteure zusammen zubringen (z. B. Horlacher und Hess 2016). Um den eigenen Bedarf nach einem CDO festzustellen und die Position im Unternehmen zu verankern, ist es für Unternehmen jedoch unerlässlich, die Unternehmensziele und strategischen Schwerpunkte festzulegen. Dies hilft ebenfalls beim Formulieren der Aufgaben und den Anforderungen an den jetzige oder zukünftige CDO (Singh et al. 2020; siehe Tab. 2 und 3 und 4).

Walchshofer und Riedl (2017) halten jedoch auch fest, dass es noch unklar ist, wohin sich die CDO-Position entwickeln wird und schlagen daher vor, diese Thematik weiter zu beforschen. Manche Wissenschaftler argumentierten, dass die zukünftige Rolle des CDOs auch vom Aufgabenfeld und der Integration des CDOs im Unternehmen abhängt. So argumentieren Haffke et al. (2016), dass CDOs, welche über den Prozess der digitalen Transformation sowie ihre Risiken und Chance aufklären, eine temporäre Rolle innehaben. Die Wissenschaftler argumentieren aber weiter, dass CDOs, welche die Initiativen zur digitalen Transformation koordinieren und Markttrends analysieren, auch langfristig benötigt werden (Haffke et al. 2016). In diesem Zusammenhang weisen Tumbas et al. (2018) darauf hin, dass, nur wenn der CDO gut im Unternehmen integriert ist und eng mit anderen Unternehmensabteilungen zusammenarbeitet, die CDO-Position auch langfristig bestehen bleiben kann. Des Weiteren könnten CDOs auch längerfristig in andere C‑Level-Positionen – bis hin zum CEO – aufsteigen (z. B. Grossman 2012).

Schließlich können Unternehmen Schwierigkeiten haben, zwischen den Positionen CIO und CDO zu unterscheiden (z. B. Walchshofer und Riedl 2017). Wenn Unternehmen jedoch den CDO als Strategen und den CIO als Verantwortlichen für die IT-Infrastruktur betrachten, wird es möglich, dass beide eng zusammenarbeiten um die digitale Transformation des Unternehmens sicherzustellen (z. B. Haffke et al. 2016). Tumbas et al. (2017) stellen jedoch fest, dass es nicht nötig ist, einen CDO einzustellen, wenn der CIO bereits einen Weg gefunden hat, neben dem Management der IT-Infrastruktur auch aktiv digitale Innovationen voranzutreiben. Zusammenfassend bleibt also festzuhalten, dass die digitale Transformation im Unternehmen eine Schlüsselrolle einnehmen sollte. Wie die damit einhergehenden Veränderungen im Unternehmen implementiert werden, hängt von vielen internen und externen Faktoren ab, die wir unter anderem in diesem Beitrag vorgestellt haben. Tumbas et al. (2017) halten ebenfalls zusammenfassend fest, dass die digitale Transformation im Unternehmen wichtig ist. Wer sich der digitalen Transformation annimmt – entweder bereits existierende Chief Marketing Officer, CIO oder eben der CDO – bleibt jedoch dem Unternehmen überlassen (Tumbas et al. 2017). Wichtig ist jedoch, dass für den jeweiligen zuständigen C‑Level-Manager entsprechende KPIs festgelegt werden, um eine Priorisierung von Projekten zur digitalen Transformation sicherzustellen (z. B. Seeher et al. 2020; siehe Tab. 4, Forschungsfrage 4).

## Fazit und Einschränkungen dieser Literaturanalyse

Die Einschränkungen des Erkenntnisanspruchs dieser Literaturanalyse sind hauptsächlich auf den Literatursuch- und Kategorisierungsprozess zurückzuführen. Bewusst wurden nur Beiträge in die Analyse miteinbezogen, die sich als Hauptthema mit der Rolle des CDOs beschäftigen. Basierend darauf wurden auch die Suchwörter und die durchsuchten Quellen festgelegt. Es kann nicht ausgeschlossen werden, dass relevante Beiträge mit anderen Suchwörtern oder in anderen Quellen gefunden worden wären. Es ist jedoch unwahrscheinlich, dass ein oder wenige nicht identifizierte Beiträge die Kernergebnisse des vorliegenden Beitrags verändern würden. Darüber hinaus impliziert die wiederholte Nennung derselben Kategorie in unterschiedlichen Beiträgen nicht zwingend die Wichtigkeit dieser Kategorie.

Ein CDO ist für die digitale Transformation im Unternehmen zuständig und – so wie die digitale Transformation – wird auch diese Rolle von vielen Faktoren beeinflusst (z. B. neuen Technologien, digitale Wettbewerber und ein sich ständig änderndes Kundenverhalten, Verhoef et al. 2021). Damit Unternehmen die CDO-Position optimal ein- und ausrichten können, wurden im Beitrag 24 Artikel analysiert, um die wesentlichen Erkenntnisse zusammenzufassen. Die Tab. 1 und 2 und 3 geben Aufschluss darüber, mit welcher Häufigkeit (i) Gründe für die Schaffung einer CDO-Position, (ii) Aufgaben eines CDO und (iii) Anforderungen an einen CDO in der Fachliteratur benannt werden. Der am meisten genannte Grund für Unternehmen, einen CDO einzustellen, ist dabei die strategische Führung bei Veränderungen. Die meistgenannte Aufgabe des CDOs ist die Digitalisierung und das Implementieren der digitalen Transformation. Damit einhergehend war die meistgenannte Anforderung an den CDO das strategische und geschäftsorientierte Denken. Neben den bisher genannten Bereichen, die sich ausschließlich auf die Rolle eines CDO beziehen, wurde auch die Abgrenzung zwischen CDO und CIO diskutiert. Basierend auf den Erkenntnissen wurden Handlungsempfehlungen für die Praxis abgeleitet (siehe Tab. 4). Somit kann diese Arbeit als Informationsquelle rund um den Einsatz und die Ausrichtung der CDO-Rolle dienen. Unternehmen können auf der Basis des aktuellen Forschungsstands individuell ableiten, ob das Einrichten einer CDO-Position zweckmäßig ist, welche Aufgaben der CDO übernehmen sollte, welche Anforderungen der CDO erfüllen sollte und wie diese neu-geschaffene Position – auch unter Berücksichtigung eines existierenden CIO – im Unternehmen ausgerichtet werden könnte.

## Anhang

**Tab. 5:** In der Literaturanalyse berücksichtigte Artikel (n. a. = nicht angegeben)

Autor	Land	Methode	Stichprobengröße
Artemenko (2020)	n. a.	Konzeptionell	n. a.
Becker et al. (2018)	n. a.	Multiple Fallstudien auf der Basis von CDO-Interview	16 Interviews
Berman et al. (2020)	23 Ländern	Umfrage	1500 Teilnehmer (Business Executives) – inklusive 750 CDOs – aus 18 Industrien
Buchwald und Lorenz (2020)	n. a.	Konzeptionell	n. a.
Ctarino et al. (2018)	Brasilien, Niederlande, Portugal	Umfrage	15 Teilnehmer
Danilova et al. (2022)	Norwegen	Längsschnittstudie, 2 Interviewrunden mit CDOs	1 Interview-Runde (2016–2017): 20 Teilnehmer 2 Interview-Runde (2019–2020): 14 Teilnehmer
Doonan (2018)	n. a.	Interviews mit Senior Digital und Workforce Technology Executives	100 Teilnehmer
Drechsler et al. (2019)	n. a.	Event Study (Event: Benennung eines CDOs und die darauffolgende Aktienwertveränderungen des Unternehmens)	101 Benennungen von CDOs
Firk et al. (2021)	Vereinigte Staaten von Amerika und Europa	Auswertung von Panel-Daten	Längsschnitt- und internationale Stichprobe von „S&P 500“-Firmen und des MSCI Europe Indexes, 7318 Firmenjahre von 913 Firmen analysiert
Haffke et al. (2016)	Europa	Interviews mit CIOs und anderen Business Executives	19 Firmen
Horlacher und Hess (2016)	n. a.	Multiple Fallstudien auf der Basis von CDO-Interviews	4 Fallstudien (Einzelhandel, Tourismus, Publishing, Bildung)
Horlacher et al. (2016)	n. a.	Multiple Fallstudien auf der Basis von Interviews mit CIOs, Chief Technology Officers und Leiter der IT-Abteilung; Analyse von Sekundärdaten wie Pressemitteilungen	2 Fallstudien (Einzelhandel, Marktforschung und Consulting), 7 Interviews
Kunisch et al. (2022)	Vereinigte Staaten von Amerika	Analyse von Archivdaten	Archivdaten-Analyse von allen „S&P 1500“-Firmen im Zeitraum 2000 bis 2018
Reck und Fliaster (2019)	Deutschland, Österreich, Schweiz	Umfrage mit Führungskräften im Digitalbereich	211 Teilnehmer
Singh und Hess (2017)	n. a.	Multiple Fallstudien auf der Basis von Interviews	6 Fallstudien (Publishing, Finanz-Industrie, Marktforschung, Bildung, Tourismus, Einzelhandel), 10 Interviews
Singh et al. (2020)	Europa und weltweit tätige Firmen	Multiple Fallstudien anhand von Interviews mit CDOs und deren Arbeitskollegen; Sekundardaten-Analyse wie Pressemitteilungen	4 Fallstudien (Einzelhandel, Publishing, Marktforschung und Consulting, Finanz-Services), 9 Interviews
Suheimat et al. (2018)	Weltweit tätige Werbeagenturen	Multiple Fallstudien auf der Basis von CDO-Interviews, Analyse von Sekundärdaten wie Pressemitteilungen	3 Fallstudien (Werbeagenturen)
Tahvanainen und Luoma (2018)	Nordische Länder	CDO-Interviews	10 Interviews
Tumbas et al. (2017)	n. a.	CDO-Interviews	35 Interviews
Tumbas et al. (2018)	n. a.	CDO-Interviews	35 Primär-Interviews (und 3 Follow-Up-Interviews), 1 weiteres Experteninterview (mit dem Gründer einer CDO-Community)
Wade und Obwegeser (2019)	28 Länder	Analyse von CDO-Karrieren, Umfragen mit CDOs und anderen Führungskräften im Digitalbereich, CDO-Interviews	Analyse von 508 CDO-Karrieren, über 1000 Umfrageantworten, 42 Interviews
Walchshofer und Riedl (2017)	Deutschland, Österreich, Schweiz	Analyse von Stelleninseraten, CDO-Interviews	13 Stellenausschreibungen, 6 Interviews
Zhan und Mu (2016)	Vereinigte Staaten von Amerika	Event Studie (Event: Benennung eines CDOs und die darauffolgende Aktienwertveränderungen des Unternehmens)	97 Benennungen von CDOs
Zhan et al. (2020)	n. a.	Event Studie (Event: Benennung eines CDOs und die darauffolgende Aktienwertveränderungen des Unternehmens)	112 Benennungen von CDOs

## Footnotes

1 Die Position des CDOs als Chief Digital Officer ist nicht zu verwechseln mit der Position des Chief Data Officer, eine Führungsposition, die sich mit Daten als strategische Unternehmensressource befasst (Xu et al. 2016). Auch für diese Position wird CDO als Akronym verwendet.

2 Wir haben uns dagegen entschieden „CDO“ als Suchbegriff zu verwenden, da diese Abkürzung auch für verschiedenste andere Abkürzungen steht (zum Beispiel „Collateralized Debt Obligations“, die Abkürzung steht auch für gewisse Nanokomposite). CDO steht des Weiteren für den Chief Data Officer, welcher nicht Gegenstand dieser Arbeit ist. Wir gehen davon aus, dass sich Autoren von wissenschaftlichen Studien in ihren Veröffentlichungen nicht nur auf die Abkürzung CDO beschränken, sondern auch den nicht abgekürzten Titel verwenden.

## References

[CR1] Alatovic T, Chhaya M, Juneja S et al (2020) Driving digital change during a crisis: the chief digital officer and Covid-19. McKinsey Digit, S 1–6

[CR2] Artemenko E (2020) The roles of top management in digital transformation. IOP Conf Ser Mater Sci Eng 940:12014. 10.1088/1757-899X/940/1/012014

[CR3] Becker L, Jaakkola E (2020) Customer experience: fundamental premises and implications for research. J Acad Mark Sci 48:630–648. 10.1007/s11747-019-00718-x

[CR4] Becker W, Schmid O, Botzkowski T (2018) Role of CDOs in the digital transformation of SMEs and LSEs. An empirical analysis. In: Hawaii international conference on system sciences proceedings Hilton Waikoloa Village, S 4534–4543

[CR5] Berman S, Baird CH, Eagan K, Marshall A (2020) What makes a chief digital officer successful? Strateg Leadersh 48:32–38. 10.1108/SL-12-2019-0180

[CR50] vom Brocke J, Simons A, Niehaves B et al (2009) Reconstructing the giant: On the importance of rigour in documenting the literature search process. In: European Conference on Information Systems Proceedings Verona, S 2206–2217

[CR6] Buchwald A, Lorenz F (2020) Who is in charge of digital transformation? The birth and rise of the chief digital officer. Acad Manag Proc 2020:14317. 10.5465/ambpp.2020.14317abstract

[CR7] Ctarino J, Rosa I, Da Silva MM (2018) Defining the chief digital officer using COBIT5. ISACA J 6:41–48

[CR8] Danilova K, Iden J, Bygstad B (2022) Chief digital officers’ evolving strategies: balancing lightweight and heavyweight IT during the digital transformation. In: Hawaii International Conference on System Sciences Proceedings, S 6403–6412

[CR9] Doonan M (2018) So you’ve just hired a killer chief digital officer—now what? Strateg HR Rev 17:17–22. 10.1108/shr-11-2017-0073

[CR10] Drechsler K (2020) Information systems executives : a review and research agenda. In: European Conference on Information Systems Proceedings, S 1–16

[CR11] Drechsler K, Wagner HT, Reibenspiess V (2019) Risk and return of chief digital officers’ appointment—An event study. In: International Conference on Information Systems Proceedings Munich, S 1–17

[CR12] Eckles D, Gordon BR, Johnson GA (2018) Field studies of psychologically targeted ads face threats to internal validity. Proc Natl Acad Sci 115:E5254–E5255. 10.1073/pnas.1805363115 29777091 10.1073/pnas.1805363115 PMC6003331

[CR13] Firk S, Hanelt A, Oehmichen J, Wolff M (2021) Chief digital officers: An analysis of the presence of a centralized digital transformation role. J Manag Stud 58:1800–1831. 10.1111/joms.12718

[CR14] Fitzgerald M, Kruschwitz N, Bonnet D, Welch M (2013) Embracing digital technology: A new strategic imperative. MIT Sloan Manag Rev. https://sloanreview.mit.edu/projects/embracing-digital-technology/. Zugegriffen: 31.3.2022

[CR15] Gentile C, Spiller N, Noci G (2007) How to sustain the customer experience: an overview of experience components that co-create value with the customer. Eur Manag J 25:395–410. 10.1016/j.emj.2007.08.005

[CR16] Giebe C (2019) The chief digital officer: Savior for the digitalization in german banks? J Econ Dev Environ People 8:6. 10.26458/jedep.v8i3.633

[CR17] Grewal D, Roggeveen AL (2020) Understanding retail experiences and customer journey management. J Retail 96:3–8. 10.1016/j.jretai.2020.02.002

[CR18] Grossman R (2012) The rise of the chief digital officer. https://www.russellreynolds.com/en/insights/reports-surveys/the-rise-of-the-chief-digital-officer. Zugegriffen: 1. Dez. 2021

[CR19] Haffke I, Kalgovas B, Benlian A (2016) The role of the CIO and the CDO in an organization’s digital transformation. In: International Conference on Information Systems Proceedings Dublin, S 1–20

[CR20] Hermes A, Riedl R (2020) The nature of customer experience and its determinants in the retail context: Literature review. In: Gronau N, Heine M, Krasnova, Pousttchi K (Hrsg) WI2020 Zentrale Tracks. GITO, Potsdam, S 1738–1749

[CR21] Hermes A, Riedl R (2021a) Dimensions of retail customer experience and its outcomes: A literature review and directions for future research. In: Nah FFH, Siau K (Hrsg) Lecture Notes in Computer Science—HCI in Business, Government and Organizations. Springer, Cham, S 71–89

[CR22] Hermes A, Riedl R (2021b) Influence of personality traits on choice of retail purchasing channel: literature review and research agenda. J Theor Appl Electron Commer Res 16:3299–3320. 10.3390/jtaer16070179

[CR23] Hess T, Matt C, Benlian A, Wiesböck F (2016) Options for formulating a digital transformation strategy. Mis Q Exec 15:103–119

[CR24] Hirsh JB, Kang SK, Bodenhausen GV (2012) Personalized persuasion. Psychol Sci 23:578–581. 10.1177/0956797611436349 22547658 10.1177/0956797611436349

[CR25] Horlacher A, Hess T (2016) What does a chief digital officer do? Managerial tasks and roles of a new c‑level position in the context of digital transformation. In: Hawaii international conference on system sciences proceedings Koloa, S 5126–5135

[CR26] Horlacher A, Klarner P, Hess T (2016) Crossing boundaries: organization design parameters surrounding CDos and their digital transformation activities. In: Americas conference on information systems proceedings San Diego. S 1–10

[CR27] Kessel L, Graf-Vlachy L (2021) Chief digital officers: the state of the art and the road ahead. Manag Rev Q. 10.1007/s11301-021-00227-8

[CR28] Kunisch S, Menz M, Langan R (2022) Chief digital officers: an exploratory analysis of their emergence, nature, and determinants. Long Range Plann 55:101999. 10.1016/j.lrp.2020.101999

[CR29] Lemon KN, Verhoef PC (2016) Understanding customer experience throughout the customer journey. J Mark 80:69–96. 10.1509/jm.15.0420

[CR30] Lim HS, Bouchacourt L, Brown-Devlin N (2021) Nonprofit organization advertising on social media: the role of personality, advertising appeals, and bandwagon effects. J Consum Behav 20:849–861. 10.1002/cb.1898

[CR31] Mani R (2017) Is chief digital officer (CDO) just a fad? https://www.dynamiccio.com/is-chief-digital-officer-cdo-just-a-fad/. Zugegriffen: 30. Nov. 2021

[CR32] Moker A (2020) What do we know about the chief digital officer ? A literature review. In: Americas conference on information systems proceedings, S 1–5

[CR33] Page MJ, McKenzie JE, Bossuyt PM et al (2021) The PRISMA 2020 statement: an updated guideline for reporting systematic reviews. BMJ. 10.1136/bmj.n71 10.1136/bmj.n71 PMC8005924 33782057

[CR34] Reck F, Fliaster A (2019) Four profiles of successful digital executives four types of effective digital executives in business organizations. Mit Sloan Manag Rev 60:1–7

[CR35] Riedl R (2019) Management von Informatik-Projekten: Digitale Transformation erfolgreich gestalten, 2. Aufl. De Gruyter Oldenbourg, München

[CR36] Scott ML, Martin KD, Wiener JL et al (2020) The Covid-19 pandemic at the intersection of marketing and public policy. J Public Policy Mark 39:257–265. 10.1177/0743915620932151

[CR37] Seeher V, Beimborn D, Holotiuk F (2020) How to evaluate the performance of the chief digital officer: a delphi study on KPis for CDos. In: European conference of information systems proceedings, S 1–15

[CR38] Sheth J (2020) Impact of Covid-19 on consumer behavior: will the old habits return or die? J Bus Res 117:280–283. 10.1016/j.jbusres.2020.05.059 32536735 10.1016/j.jbusres.2020.05.059 PMC7269931

[CR39] Singh A, Hess T (2017) How chief digital officers promote the digital transformation of their companies. Mis Q Exec 16:1–17. 10.4324/9780429286797-9

[CR40] Singh A, Klarner P, Hess T (2020) How do chief digital officers pursue digital transformation activities? The role of organization design parameters. Long Range Plann 53:101890. 10.1016/j.lrp.2019.07.001

[CR41] Stieninger M, Auinger A, Riedl R (2019) Digitale Transformation im stationären Einzelhandel. Wirtschaftsinform Manag 11:46–56. 10.1365/s35764-018-0152-4

[CR42] Suheimat W, Prætorius T, Vang J (2018) Building dynamic capabilities in large global advertising agency networks: managing the shift from mass communication to digital interactivity. Int J Foresight Innov Policy 13:88–113. 10.1504/IJFIP.2018.095860

[CR43] Tahvanainen S, Luoma E (2018) Examining the competencies of the chief digital officer. In: Americas conference on information systems proceedings New Orleans, S 1–10

[CR45] Tumbas S, Berente N, vom Brocke J (2017) Three types of chief digital officers and the reasons organizations adopt the role. Mis Q Exec 16:121–134. 10.4324/9780429286797-14

[CR44] Tumbas S, Berente N, vom Brocke J (2018) Digital innovation and institutional entrepreneurship: chief digital officer perspectives of their emerging role. J Inf Technol 33:188–202. 10.1057/s41265-018-0055-0

[CR46] Van Looy A (2021) How the Covid-19 pandemic can stimulate more radical business process improvements: using the metaphor of a tree. Knowl Process Manag 28:107–116. 10.1002/kpm.1659

[CR47] Verband der Hochschullehrer für Betriebswirtschaft e. V. (2019) VHB-JOURQUAL 3. https://vhbonline.org/vhb4you/vhb-jourqual/vhb-jourqual-3. Zugegriffen: 28. Febr. 2022

[CR48] Verhoef PC, Broekhuizen T, Bart Y et al (2021) Digital transformation: a multidisciplinary reflection and research agenda. J Bus Res 122:889–901. 10.1016/j.jbusres.2019.09.022

[CR49] Vial G (2019) Understanding digital transformation: a review and a research agenda. J Strateg Inf Syst 28:118–144. 10.1016/j.jsis.2019.01.003

[CR51] Wade M, Obwegeser N (2019) How to choose the right digital leader for your company. Mit Sloan Manag Rev 60:1–9

[CR52] Walchshofer M, Riedl R (2017) Der Chief Digital Officer (CDO): Eine empirische Untersuchung. HMD Prax Wirtschaftsinformatik 54:324–337. 10.1365/s40702-017-0320-7

[CR53] Webster J, Watson RT (2002) Analyzing the past to prepare for the future: Writing a literature review. Mis Q 26:xiii–xxiii

[CR54] Wedel M, Kannan PK (2016) Marketing analytics for data-rich environments. J Mark 80:97–121. 10.1509/jm.15.0413

[CR55] Xu F, Zhang H, Huang W et al (2016) The value of chief data officer presence on firm performance. In: Pacific Asia conference on information systems proceedings

[CR56] Zhan X, Mu Y (2016) Examining the shareholder value effects of announcements of CDO positions. In: International conference on service systems and service management proceedings Kunming, S 1–6

[CR57] Zhan X, Mu Y, Nishant R, Singhal VR (2020) When do appointments of chief digital or data officers (CDOs) affect stock prices. IEEE Trans Eng Manag. 10.1109/TEM.2020.2984619

